# Distribution of Cortical Diffusion Tensor Imaging Changes in Multiple Sclerosis

**DOI:** 10.3389/fphys.2020.00116

**Published:** 2020-03-13

**Authors:** Benjamin Stock, Manoj Shrestha, Alexander Seiler, Christian Foerch, Elke Hattingen, Helmuth Steinmetz, Ralf Deichmann, Marlies Wagner, René-Maxime Gracien

**Affiliations:** ^1^Department of Neurology, Goethe University Frankfurt, Frankfurt, Germany; ^2^Department of Neuroradiology, Goethe University Frankfurt, Frankfurt, Germany; ^3^Brain Imaging Center, Goethe University Frankfurt, Frankfurt, Germany

**Keywords:** diffusion tensor imaging, mean diffusivity, fractional anisotropy, cortex, gray matter, multiple sclerosis

## Abstract

**Purpose:**

Diffuse cortical damage in relapsing–remitting multiple sclerosis (RRMS) is clinically relevant but cannot be directly assessed with conventional MRI. In this study, it was aimed to use diffusion tensor imaging (DTI) techniques with optimized intrinsic eddy current compensation to quantify and characterize cortical mean diffusivity (MD) and fractional anisotropy (FA) changes in RRMS and to analyze the distribution of these changes across the cortex.

**Materials and Methods:**

Three-Tesla MRI acquisition, mapping of the MD providing information about the integrity of microstructural barriers and of the FA reflecting axonal density and surface-based analysis with Freesurfer were performed for 24 RRMS patients and 25 control subjects.

**Results:**

Across the whole cortex, MD was increased in patients (*p* < 0.001), while surface-based analysis revealed focal cortical FA decreases. MD and FA changes were distributed inhomogeneously across the cortex, the MD increase being more widespread than the FA decrease. Cortical MD correlated with the Expanded Disability Status Scale (EDSS, *r* = 0.38, *p* = 0.03).

**Conclusion:**

Damage of microstructural barriers occurs inhomogeneously across the cortex in RRMS and might be spatially more widespread than axonal degeneration. The results and, in particular, the correlation with the clinical status indicate that DTI might be a promising technique for the monitoring of cortical damage under treatment in larger clinical studies.

## Introduction

Multiple sclerosis (MS) is an inflammatory disease of the central nervous system (CNS), characterized by typical focal lesions, but also by diffuse tissue changes. While conventional magnetic resonance imaging (MRI) techniques are essential for the visualization of the lesion load and, accordingly, for the initial diagnosis of MS, quantitative MRI (qMRI) techniques also allow for the assessment of inconspicuous changes in tissue composition (Cercignani et al., 2018). Different qMRI techniques such as T1 and T2 relaxometry and proton density (PD) mapping have been applied in MS (Vrenken et al., 2006a; Gracien et al., 2016). Another promising qMRI method in MS is diffusion tensor imaging (DTI), providing the advantage of a widespread availability on standard clinical systems. The DTI parameter mean diffusivity (MD) describes the amount of diffusion-related movement of water molecules, whereas the fractional anisotropy (FA) provides information about the directionality of diffusion (Cercignani et al., 2018). While macroscopic white matter (WM) lesions can be visualized with conventional MRI techniques, assessment of the gray matter (GM) and, particularly, of cortical changes is more challenging. Still, cortical damage seems to have a high impact on clinical symptoms observed in MS patients and likely contributes to fatigue, depression, and cognitive impairment in MS (Calabrese et al., 2009; Gobbi et al., 2014). DTI has the potential to provide insights into cortical changes in MS. However, previous DTI investigations on MS patients revealed contradictory results. Several studies have observed increased MD in the GM (Ceccarelli et al., 2007; Rovaris et al., 2008; Yu et al., 2008) and, in particular, in the cerebral cortex of MS patients (Hasan et al., 2012), likely indicating a loss of microstructural barriers, while other studies reported no MD differences in the GM as compared to healthy subjects (Griffin et al., 2001; Tortorella et al., 2006). Similarly, for FA, some studies found increased values in the GM (Calabrese et al., 2011) and in the cortex of MS patients (Hasan et al., 2012), while other studies reported decreased cortical FA values (Vrenken et al., 2006b), potentially reflecting axonal degeneration, or no FA changes in the GM (Griffin et al., 2001; Tortorella et al., 2006). Furthermore, so far, it is unclear how MD and FA changes distribute across the cortex.

In summary, in light of the previous results, the questions arise whether cortical FA and MD values are suited to characterize cortical remodeling in MS and, if so, whether cortical FA and MD values are increased or decreased in MS, how alterations of diffusion parameters distribute across the cortex, and how spatial distributions of parameter changes relate to each other.

In the present study, it was aimed to investigate these questions, using an optimized DTI sequence with intrinsic eddy current compensation combined with a surface-based cortical Freesurfer analysis. In particular, considering that some previous studies yielded contradictory or negative results, it was intended to demonstrate and confirm the presence of cortical MD and FA changes reflecting cortical damage in MS and to quantify and characterize these abnormalities, hypothesizing that cortical MD reflecting the integrity of microstructural barriers might be increased and FA quantifying axonal density might be decreased in MS. Furthermore, it was aimed to assess and compare the spatial distributions of cortical FA and MD changes. For this purpose, an optimized DTI sequence was used with intrinsic compensation of eddy current effects, thus reducing a potential bias which might otherwise increase the variability of DTI parameter values across the group and render detection of MD and FA changes more difficult.

## Materials and Methods

### Participants

Twenty-four patients with relapsing–remitting MS (RRMS, 11 male) and 25 matched healthy control subjects (11 male) participated in the study. Patients were examined and rated on the Expanded Disability Status Scale (EDSS) (Kurtzke, 1983). The cohort overlaps with a previous study using relaxometry and PD mapping in MS (van Wijnen et al., 2019). However, the present study has different aims and investigates changes of DTI parameters. The studies involving human participants were reviewed and approved by the respective local board (Ethik-Kommission des Fachbereichs Medizin des Universitätsklinikums der Goethe-Universität). The patients/participants provided written informed consent to participate in this study. The study was performed according to the principles formulated in the Declaration of Helsinki.

### Data Acquisition and Processing

MRI data acquisition was performed on a 3-Tesla (T) whole-body magnetic resonance (MR) scanner (Trio, Siemens Healthineers, Erlangen, Germany) which uses a body coil for radio-frequency (RF) transmission and an 8-channel phased-array head coil for RF reception.

For data processing and analysis, functions from Freesurfer 6.0.1 (Dale et al., 1999; Fischl et al., 1999) and from the FMRIB Software Library 5.0.7 (FSL) (Smith et al., 2004) were used.

**FIGURE 1:**
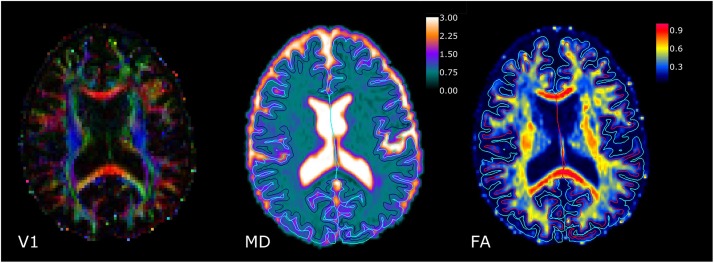
Results of diffusion tensor imaging data processing (single slice, representative patient). Left: Map of the direction of the diffusion tensor’s principal eigenvector (V1). The luminosity denotes the fractional anisotropy (FA) value. Middle: Map of the mean diffusivity (MD). Right: Map of FA. MD is given in units of 10^–3^ mm^2^ s^–1^. In the MD and FA maps, the light blue lines indicate the pial surface, and the dark blue/red lines the white matter surface, as identified with Freesurfer.

DTI data were acquired using a diffusion-weighted (DW) twice-refocused (tr) spin-echo (SE) echo planar imaging (EPI) sequence (Heid, 2000; Reese et al., 2003) with the optimizations described previously in the literature (Shrestha et al., 2018). A schematic description of this method was presented by Heid (2000) and by Reese et al. (2003) (Figure 1 in both cases). A pulse diagram of the sequence as used in the present study can be found in Figure 1B in the publication describing the optimizations (Shrestha et al., 2018). These include: (1) Assumption of an eddy-current decay time of 40 ms. This value was chosen individually for the MR system used on the basis of preliminary tests. (2) Insertion of crusher gradients around the refocusing pulses, allowing for complete spoiling of the transverse magnetization for the chosen voxel size. These spoiler gradients are especially required for obtaining artifact-free base images with *b* = 0. (3) Usage of symmetrically distributed DW gradient (DWG) directions for full-sphere sampling. Protocol parameters used to acquire DTI data were: field of view (FoV) = 192 × 192 mm^2^, isotropic resolution = 2 mm, 70 interleaved axial slices without interslice gap, TR = 9,300 ms, TE = 95 ms, echo-spacing = 0.86 ms, readout bandwidth = 1,302 Hz/pixel, partial Fourier = 25%, two-fold acceleration, 60 different diffusion encoding directions at *b*-value = 1,000 s/mm^2^ (with DWG amplitude of 28 mT/m). In order to correct for geometrical distortions induced by static magnetic field (B0) inhomogeneities, two sets of five reference images with *b* = 0 were acquired with either positive or negative phase encoding gradients (i.e., traversing k-space in different directions), yielding five pairs of DTI data with opposite distortions. These data were processed using TOPUP to estimate the susceptibility-induced off-resonance field (Andersson et al., 2003), and DTI data were subsequently corrected for these effects. Brain extraction was performed with BET (Smith, 2002). Correction for residual eddy-current-induced distortions and for subject movement was performed for voxels inside the resulting brain mask with EDDY using standard parameters (Andersson and Sotiropoulos, 2016).

For segmentation purposes, optimized synthetic MP-RAGE anatomies which are intrinsically corrected for bias fields (Gracien et al., 2019) were derived from qMRI maps of T1, B0, and the transmitted field B1 as described previously in the literature (Gracien et al., 2019), assuming the following virtual MP-RAGE acquisition parameters: FoV = 256 × 224 × 160 mm^3^, isotropic resolution = 1 mm, TR = 1,900 ms, TI = 900 ms, α = 9°. The total acquisition time for all underlying qMRI maps was 16:42 min.

### Data Analysis

The recon-all stream implemented in the Freesurfer toolbox (Dale et al., 1999; Fischl et al., 1999) was applied to the synthetic MP-RAGE anatomies for cortical segmentation and for vertex-wise measurement of the cortical thickness. Boundary-based coregistration of the MD maps to the MP-RAGE anatomies was performed using BBREGISTER (Greve and Fischl, 2009). Using the respective coregistration matrices, cortical FA and MD values were read, averaged for each vertex, and saved in surface datasets. Accordingly, in this step, the parameter values across the whole cortex were stored in surface datasets with a high resolution (∼1 mm distance between the vertices). After normalization (“fsaverage space”) of these datasets and the cortical thickness maps and smoothing (Gaussian kernel with a full width at half maximum of 10 mm), a general linear model analysis was carried out for two-tailed statistical comparison between groups, including correction for multiple comparisons *via* Monte Carlo simulation.

Furthermore, cortical non-zero FA and MD values were averaged across the surface datasets, and average values were compared between groups with multivariate analysis of variance (MANOVA). Correction for multiple comparisons was performed for the respective tests using the false discovery rate (FDR) method/Benjamini–Hochberg procedure (FDR = 0.05). Additionally, mean cortical thickness values were compared between patients and healthy subjects *via* a two-tailed *t*-test.

To analyze different anatomical cortical regions of interest (ROIs), the cortex atlas “PALS_B12_Lobes” was coregistered to each subject with mri_surf2surf, and cortical MD/FA values were read vertex-wise and averaged for the frontal, temporal, parietal, and occipital lobes. Since surface-based analysis revealed symmetric patterns of DTI changes, parameter values in the ROIs averaged across both hemispheres were compared between groups *via* two-tailed *t*-tests. Furthermore, tests for Spearman’s rank-order correlations between cortical parameters with significant group differences and the EDSS were carried out using one-tailed tests for significance and assuming that pathological cortical changes might correlate with clinical deficits. *P* values below 0.05 were considered significant.

## Results

The average age (± standard deviation, SD) of the participants was 35.4 ± 10.6 years for patients and 34.5 ± 11.1 years for healthy subjects. Age did not differ between groups (*p* = 0.77). Average EDSS scores of the patients amounted to 2.9 ± 1.7 (range 0–9), and disease durations were 8.0 ± 5.8 years (range 2–29 years).

Six patients were treated with dimethyl fumarate, five patients with natalizumab, five with fingolimod, three with glatiramer acetate, two with interferon beta, and one with rituximab. Two patients were untreated.

Figure 1 demonstrates for a single slice and a representative patient the results of DTI data processing, showing (from left to right) a map of the direction of the diffusion tensor’s principal eigenvector (V1) where the luminosity denotes the FA value, an MD map, and an FA map. In the MD and FA maps, the light blue lines indicate the pial surface, and the dark blue/red lines the WM surface, as identified with Freesurfer.

**FIGURE 2:**
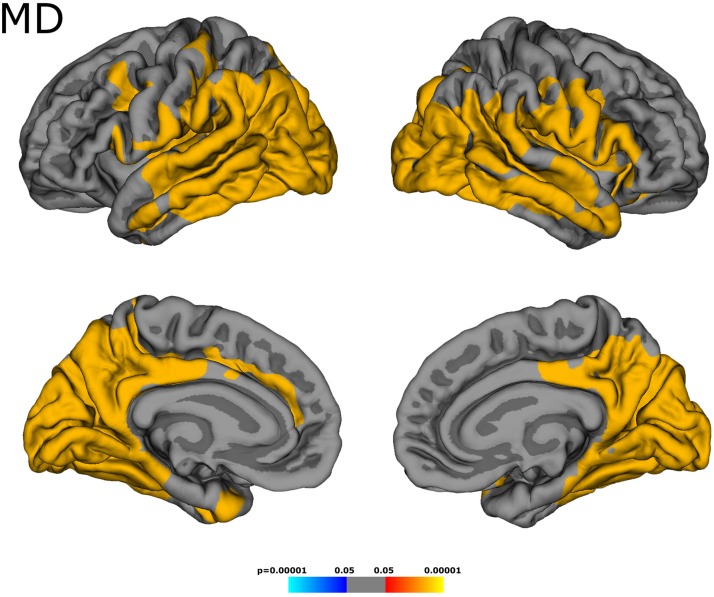
Cortical areas with increased mean diffusivity (MD). Spatially inhomogeneous MD changes were mainly located in temporal, occipital, and some parietal regions.

**FIGURE 3:**
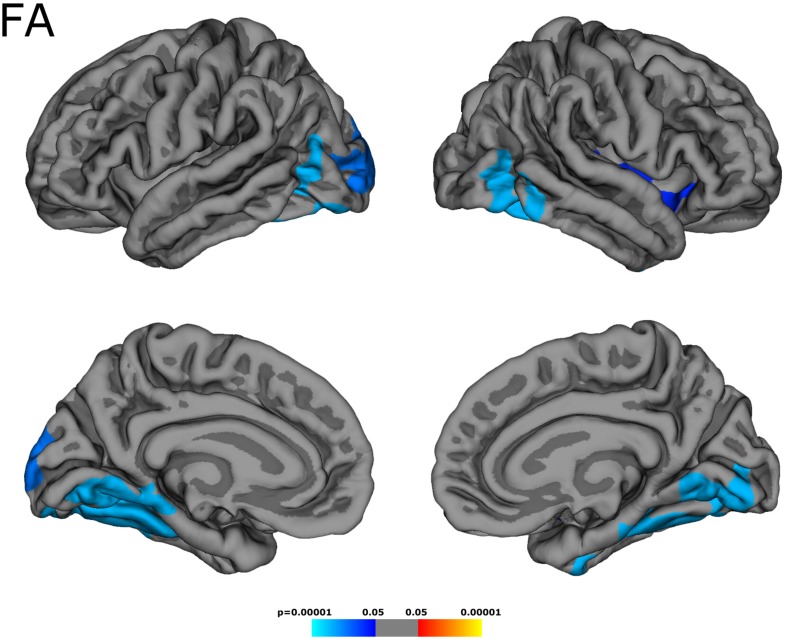
Cortical areas with decreased fractional anisotropy (FA). Focal FA changes were mostly observed in temporo-occipital regions.

Across the whole cortex, the group had a significant effect on MD/FA values [F (2, 46) = 8.03, *p* = 0.001]. MD was higher in patients than in control subjects (patients: 0.969 ± 0.053 × 10^–3^ mm^2^ s^–1^, control subjects: 0.920 ± 0.029 × 10^–3^ mm^2^ s^–1^, *p* < 0.001). As demonstrated in Figure 2, cortical areas with increased MD were distributed inhomogeneously across the cortex, being mainly located in the temporal and occipital lobes and in some parietal regions. In line with these results, ROI-based analysis of cortical lobes as demonstrated in Table 1 revealed an MD increase for all lobes, with the numerically smallest difference (0.022 × 10^–3^ mm^2^ s^–1^) for the frontal cortex. While FA did not differ between groups across the whole cortex (patients: 0.165 ± 0.011, control subjects: 0.167 ± 0.006, *p* = 0.31), focal FA decreases were observed mostly in temporo-occipital regions (Figure 3 and Table 1). The cortical thickness did not differ between groups (patients: 2.43 ± 0.07 mm, control subjects: 2.44 ± 0.07 mm, *p* = 0.50), and surface-based analysis did not unveil focal changes of cortical thickness.

**TABLE 1:** Fractional anisotropy (FA) and mean diffusivity (MD) values for the different cortical lobes.

		Patients		Healthy subjects		*P*
**FA**	**Frontal cortex**	0.173	±0.009	0.171	±0.007	0.38
	**Parietal cortex**	0.154	±0.013	0.154	±0.008	0.97
	**Temporal cortex**	0.169	±0.012	0.177	±0.007	**0.009**
	**Occipital cortex**	0.143	±0.014	0.151	±0.010	**0.03**
**MD**	**Frontal cortex**	0.957	±0.030	0.935	±0.029	**0.01**
	**Parietal cortex**	1.028	±0.083	0.973	±0.038	**0.004**
	**Temporal cortex**	0.909	±0.037	0.861	±0.026	**<0.001**
	**Occipital cortex**	1.010	±0.123	0.917	±0.049	**0.001**

*Significant *p*-values are presented in bold. MD is given in units of 10^–3^ mm^2^ s^–1^.*

Furthermore, the EDSS correlated with the cortical MD (*r* = 0.38, *p* = 0.03). As the other parameters did not show significant group differences across the entire cortex, respective tests for correlation with EDSS were not performed.

## Discussion

The presented study investigates cortical changes in MS using high-resolution DTI techniques with improved intrinsic correction for eddy currents. A focal cortical FA decrease and more widespread areas with increased MD were observed. FA and MD changes distributed inhomogeneously across the cortex, indicating that cortical remodeling in MS might vary across the cortex.

The question arises which microstructural pathological changes are measured by cortical FA and MD. As FA values are high in fiber tracts, previous analyses indicate that axonal degeneration might be reflected by FA decreases in MS (Ciccarelli et al., 2003). Furthermore, since MD quantifies the diffusion-related movement of water molecules which can be limited by barriers, it has been suggested that MD provides information about the integrity of microstructural barriers (Filippi et al., 2000). In the present study, most cortical regions with decreased FA in patients also showed increased MD. However, MD increases were found to be spatially more widespread than FA decreases. These results imply that changes of microstructural barriers might affect more cortical areas than axonal loss or that MD might be the more sensitive parameter.

The cortical MD (0.97 ± 0.05 × 10^–3^ mm^2^ s^–1^) and FA values (0.17 ± 0.01) that were found for MS patients in the present investigation are within the range reported in previous studies (MD: 0.92–1.18 × 10^–3^ mm^2^ s^–1^; FA: 0.13–0.23) (Griffin et al., 2001; Tortorella et al., 2006; Vrenken et al., 2006b; Ceccarelli et al., 2007; Rovaris et al., 2008; Yu et al., 2008; Calabrese et al., 2011; Hasan et al., 2012). The observed cortical MD increases are in line with results of previous studies which demonstrated increased MD in the GM (Ceccarelli et al., 2007; Rovaris et al., 2008; Yu et al., 2008) and in the cortex (Hasan et al., 2012) in MS. In contrast, other investigations reported no MD changes in the GM (Griffin et al., 2001; Tortorella et al., 2006). FA was decreased in some cortical regions in the presented and in a previous study (Vrenken et al., 2006b), while other investigations observed increased GM (Calabrese et al., 2011) and cortical FA values (Hasan et al., 2012) or no changes in the GM (Griffin et al., 2001; Tortorella et al., 2006). It should be noted that different DTI protocols and postprocessing steps and different methods for identifying GM were used in previous studies which might explain the divergent results. In particular, some earlier studies (Griffin et al., 2001; Tortorella et al., 2006; Vrenken et al., 2006b; Rovaris et al., 2008) used DTI protocols with a slice thickness equal to or exceeding 5 mm (2 mm in the present study), which might increase the risk of partial volume effects and render cortical/GM analysis more difficult. Importantly, another explanation for some diverging findings might be that previous studies yielding negative results (Griffin et al., 2001; Tortorella et al., 2006) investigated patients with a shorter average disease duration of approximately 2 years (8 years in the present study). Therefore, it is likely that the cohorts in these previous investigations comprised patients with a lower degree of tissue changes as compared to the cohort in the presented study.

An optimized DW-trSE-EPI sequence with online eddy current compensation (Shrestha et al., 2018) was applied in the presented study. Conventional DW imaging based on a DW single-refocused SE preparation (Stejskal and Tanner, 1965; Turner et al., 1990) usually employs two identical monopolar DWGs which are placed symmetrically around a single refocusing RF pulse, followed by an EPI readout. The problem with this approach is that switching a monopolar DWG induces eddy currents, yielding image distortions that depend on the DWG direction. To overcome this problem, a DW-trSE-EPI sequence with intrinsic eddy current compensation was proposed, comprising four bipolar DWGs placed around two refocusing RF pulses (Heid, 2000; Reese et al., 2003). The method employs bipolar DWGs with identical amplitudes but different durations, thus allowing for a full correction of eddy current artifacts. Recently, this sequence was further improved by introducing resolution-dependent crusher gradients, placed around the refocusing pulses (Shrestha et al., 2018). This optimized sequence was used in the presented investigation. While DTI techniques without intrinsic eddy current compensation acquire uncorrected data in a first step and apply postprocessing subsequently to correct for eddy-current-induced distortions, the method employed here avoids such artifacts intrinsically by using bipolar DWGs with mutual cancelation of their respective eddy current contributions directly prior to the EPI readout. Therefore, this method might be useful to minimize a technical bias on the FA and MD maps, which might otherwise impose an erroneously increased variation of the respective parameter values across the investigated cohort. Thus, in light of some diverging findings in previous studies, the method might be able to yield more reliable quantifications of MD and FA changes in MS. Possibly, this might be the reason why a significant correlation of cortical MD values with the clinical status (EDSS) could be observed in the presented study.

It should be noted that DTI techniques can also provide important information about tissue damage in the spinal cord in MS (Toosy et al., 2014; Oh et al., 2015). The trSE-EPI sequence with intrinsic eddy current compensation as proposed by Reese et al. (2003) and Heid (2000) was applied in a previous study of the spinal cord in MS to demonstrate that diffusional kurtosis imaging measuring both Gaussian and non-Gaussian properties of water diffusion is able to provide complementary information to DTI (Raz et al., 2013). The combination of the trSE-EPI sequence (Heid, 2000; Reese et al., 2003) with the optimizations proposed previously (Shrestha et al., 2018), as used in the present study, might also be applicable and useful for the assessment of the spinal cord in future studies.

A limitation of the study is the application of only one DTI-acquisition method with two *b*-values (0 and 1,000 s/mm^2^). It has been shown that DTI data based on acquisitions with multiple *b*-values can provide complementary information (Peled et al., 2009). Furthermore, diffusion measurement based on several *b*-values in MS (Ciccarelli et al., 2001) might increase accuracy even though the difference between the results of two and multiple-point techniques can be expected to be small (Burdette et al., 1998). Additionally, it should be noted that the sample size was relatively small. Future studies comparing different DTI acquisition methods with multiple *b*-values and analysis techniques in a larger MS cohort might help to further elucidate the origins of the partly contradicting findings in previous DTI studies in MS.

In conclusion, the observed focal cortical FA decreases and more widespread MD increases indicate that inhomogeneously distributed clinically relevant cortical damage of microstructural barriers might locally go beyond axonal degeneration in RRMS. Furthermore, the findings suggest that DTI techniques might be helpful for the investigation of cortical damage in larger clinical studies, including trials comparing different therapy arms.

## Data Availability Statement

The datasets for this article are not available publicly or upon direct request because data sharing does not comply with the institutional ethics approval.

## Ethics Statement

The studies involving human participants were reviewed and approved by the Ethik-Kommission des Fachbereichs Medizin des Universitätsklinikums der Goethe-Universität. The patients/participants provided their written informed consent to participate in this study.

## Author Contributions

MS, RD, MW, and R-MG contributed to the conception and design of the study. BS, RD, MW, and R-MG organized the study. BS, MW, and R-MG executed the study and acquired the data. RD derived the synthetic MP-RAGE anatomies from the source data. R-MG derived the FA/MD maps from the source data, and designed and performed the statistical analysis. BS and R-MG wrote the first draft of the manuscript. All authors reviewed the statistical analysis and contributed to the manuscript revision and read and approved the submitted version.

## Conflict of Interest

HS has received speaker’s honoraria from Bayer, Sanofi, and Boehringer Ingelheim. RD received compensation as a consultant for MR scanner procurement by the Wellcome Trust Centre for Neuroimaging, UCL, London, United Kingdom. The remaining authors declare that the research was conducted in the absence of any commercial or financial relationships that could be construed as a potential conflict of interest.

## Abbreviations

CNS: central nervous system
DTI: diffusion tensor imaging
DW: diffusion-weighted
DWG: diffusion-weighted gradient
EDSS: Expanded Disability Status Scale
EPI: echo-planar imaging
FA: fractional anisotropy
FoV: field of view
FSL: FMRIB Software Library
GM: gray matter
MD: mean diffusivity
MP-RAGE: magnetization-prepared rapid gradient echo
MR: magnetic resonance
MRI: magnetic resonance imaging
MS: multiple sclerosis
PD: proton density
qMRI: quantitative magnetic resonance imaging
RF: radio-frequency
RRMS: relapsing–remitting multiple sclerosis
SD: standard deviation
SE: spin echo
T: Tesla
TE: echo time
TR: repetition time
tr: twice refocused
WM: white matter.
